# The RNA world and the origin of metabolic enzymes

**DOI:** 10.1042/BST20140132

**Published:** 2014-08-11

**Authors:** Markus Ralser

**Affiliations:** * Department of Biochemistry and Cambridge Systems Biology Centre, University of Cambridge, 80 Tennis Court Road, Cambridge CB2 1GA, U.K.; † Division of Physiology and Metabolism, MRC National Institute for Medical Research, The Ridgeway, Mill Hill, London NW7 1AA, U.K.

**Keywords:** catalysis, evolution, glycolysis, origin of life, origin of metabolism, pentose phosphate pathway, replicator, RNA

## Abstract

An RNA world has been placed centre stage for explaining the origin of life. Indeed, RNA is the most plausible molecule able to form both a (self)-replicator and to inherit information, necessities for initiating genetics. However, in parallel with self-replication, the proto-organism had to obtain the ability to catalyse supply of its chemical constituents, including the ribonucleotide metabolites required to replicate RNA. Although the possibility of an RNA-catalysed metabolic network has been considered, it is to be questioned whether RNA molecules, at least on their own, possess the required catalytic capacities. An alternative scenario for the origin of metabolism involves chemical reactions that are based on environmental catalysts. Recently, we described a non-enzymatic glycolysis and pentose phosphate pathway-like reactions catalysed by metal ions [mainly Fe(II)] and phosphate, simple inorganic molecules abundantly found in Archaean sediments. While the RNA world can serve to explain the origin of genetics, the origin of the metabolic network might thus date back to constraints of environmental chemistry. Interestingly, considering a metal-catalysed origin of metabolism gives rise to an attractive hypothesis about how the first enzymes could have formed: simple RNA or (poly)peptide molecules could have bound the metal ions, and thus increased their solubility, concentration and accessibility. In a second step, this would have allowed substrate specificity to evolve.

## Introduction

We are far from understanding the origin of life, as several of the problems associated with it are yet unsolved [1,2]. Some of the remaining questions associated with it appear, however, to be the most crucial ones. First, how did early biomolecules, supposedly nucleotides, sugars, amino acids and fatty acids, form and reach life compatible concentrations? In this context, one needs to consider how the first forms of geochemical carbon fixation could have taken place [3,4]. Moreover, as life is unlikely to have started in an extreme dilute solution, this suggests that it was, from the beginning, bound by some sort of compartmentalization [5,6].

The second question, and probably the currently better addressed one, is about how genetics, and with it inheritable evolutionary selection, could have come into place. The most plausible prevailing hypothesis for this event is the so-called RNA world. RNA would have served as a self-replicator, and in this way united the properties of a replicator that is able to inherit information while also possessing the ability to evolve [7,8]. Explaining the origin of RNA molecules on the basis of prebiotic chemistry is not simple, but is considered possible [9–11]. Supportingly, laboratory experiments have shown that RNA can form self-replicating molecules, and RNA has been used successfully in directed evolution experiments [12,13]. In addition, there are plenty of potential traces of an RNA world found in our modern cells: several viruses use RNA as their genetic material (a ‘proof of concept’ that RNA is able to act as carrier of genetic material), it can be (reversely) transcribed into DNA, and the biological synthesis pathway leading to desoxynucleotides is based on desoxylation of ribonucleotides, indicating that RNA preceded DNA. Moreover, RNA is a functional and structural component of the ribosome and DNA-modifying enzymes.

The third equally important, but least understood problem, concerns the origin of metabolism. Metabolism facilitated prototrophy of cells, or at least ecosystems, and in that way rendered them independent of specific geochemical conditions [14–16]. At least at the point when a self replicator was in place, the proto-organism had to adopt ways that ensured a constant supply of its building blocks. Without the capacity to synthesize its constituents, life would be constrained to very narrow geological conditions and unlikely to have persisted over geological timescales with their frequent changes in environment. Modern cells circumvent the dependency on geochemistry for building biomolecules via a sophisticated network of chemical reactions, known as the metabolic network. The structure of this network is highly conserved among organisms, despite chemistry that would appear to offer more possibilities of how cellular biomolecules molecules might be formed [17]. As a result, the basic design principles of the metabolic network resemble each other in all living organisms. There are two conclusions to be drawn from this observation. The first is that the metabolic network reached a state of efficiency that can barely be improved further through evolutionary selection [18–20]. The second is that, supposedly, metabolic reactions came into existence at the very early steps during evolution, and thus all present forms of metabolism descend from the same original network [1,14,17].

## There are problems in imagining an RNA-catalysed metabolic network

What molecules catalysed the reactions in the early form of the metabolic network? The possibility that RNA molecules could have served as catalysts in an early metabolic network is considered [7,8]: the RNA replicator would have adopted a way to not only self-replicate, but also to catalyse the chemical reactions that lead to the formation of its own constituents [14,15]. This hypothesis is attractive from the perspective that it would be facilitated by a simple extension of the replicator hypothesis: in that scenario the metabolic network would form stepwise, through genetic selection of ribozymes, later being replaced by protein-based enzymes [7,8,15,21]. However, there are problems associated with such a hypothesis. The most important question is whether RNA indeed possesses the catalytic capacities to form a primordial metabolic network. At least in modern organisms, RNA-catalysed reactions do not constitute metabolism. In an *in vitro* selection experiment, a ribozyme was able to catalyse the aldolase reaction, but only through recruiting a bivalent metal (to be discussed below) which acted as catalyst [22]. In contrast, RNA plays a dominant role in all steps of translation, indicating that protein biosynthesis followed RNA in evolution, and that this role was maintained [23].

Next, the question about the initial trigger is not trivial: to form an RNA nucleotide, not only one, but a series of reactions is necessary. An initial RNA-catalysed reaction system needed, in essence, to provide some sort of function, in order that genetics could select for it and improve catalysis and efficiency. In other words, evolution is selecting for the (functional) product and not for an intermediate step, so an initial RNA-based network could not have come into place one reaction at a time, but only as an already operational entity. This argument renders the origin of metabolism as an RNA-based metabolic reaction system not impossible, but substantially less probable. This problem is amplified by the notion that the least self-sustaining chemical networks lack evolvability, and evolutionary selection cannot change the chemistry in a reaction system, neither are the thermodynamic properties of metabolites subject to genetic selection [24,25]. Thus RNA genetics has its limitations in optimizing chemical reaction networks.

## A non-enzymatic origin of metabolism as an alternative

Owing to the problems of explaining the origin of metabolism in the RNA world, prebiotic precursors of metabolism were considered as an alternative [16,25,26]. We have recently found that reactions of modern cells, based on carbohydrate metabolism, glycolysis (both of the canonical Embden–Meyerhoff and the overlapping Entner–Doudoroff reaction sequence), the PPP (pentose phosphate pathway) and the Calvin cycle, possess highly similar non-enzymatic counterparts [27]. Formation of pyruvate and glucose, the end products of glycolysis/gluconeogenesis can be formed spontaneously from intermediates of glycolysis in water. In addition, metal ions such as ferrous iron and phosphate that are abundantly present in Archaean sediments [28–35], catalysed additional reactions which resembled those of the PPP [27]. The observed reactions mimic an interconversion network similar to the topology of modern glycolysis and the PPP. Importantly, despite being based on simple inorganic catalysts, the reactions occurred with substantial specificity: out of a universe of possible reaction products, after 5 h at 70°C, more than 60% of the molecules were recovered as glycolytic and PPP intermediates. These results indicate that metabolic reactions similar to those used in modern cells did not necessarily originate from the evolutionary selection of complex catalysts, but instead that they are able to occur under the chemical conditions that prevailed in the Archaean sea.

Potentially, this scenario predicts that the constitution of metabolism preceded that of genetics in the origin of life [36]. At least, this scenario provides a plausible hypothesis about the origin of the metabolic network. The carbohydrate interconversion sequences of glycolysis, the PPP and overlapping reaction cascades are centrally placed in the modern metabolic network. They provide essential precursors for amino acids, fatty acids and ribonucleotide synthesis. For the latter, the non-oxidative part of the PPP is most important. Its reactivity was largely replicated by non-enzymatic reactions that were mostly dependent on ferrous iron, Fe(II). Ferrous iron is readily water-soluble, and is assumed to have been at high concentration in the Archaean oceans [31]. It was however largely depleted from the oceans upon the great oxygenation event, as oxidized iron is largely water insoluble.

The discovery of a non-enzymatic glycolysis and PPP does not automatically solve the problem of primordial carbon fixation and does not provide a simple explanation for where the starting materials came from. Perhaps, this problem remains equally strong when considering a post-genetic origin of metabolism [4,37], or when considering carbohydrates as source for the precursors of membranes in protocells [6]. However, one has to consider that modern cells synthesize glucose and sugar phosphates in gluconeogenesis and the Calvin cycle. The discovery of non-enzymatic counterparts for glycolysis and the PPP thus open a new possibility for this problem, as they render a non-enzymatic gluconeogenesis and Calvin cycle at least possible, or, as discussed below, trigger a hypothesis on how first enzymes could have looked alike.

## Metal-ion-binding RNAs and/or peptides as early forms of enzymes?

The hypothesis of a metal-catalysed form of early metabolism opens an attractive possibility to give rise to evolution of enzymes. When Fusz et al. [22] purified a ribozyme for the aldose reaction in an *in vitro* selection experiment, they obtained an RNA molecule which bound a bivalent metal, Zn^2+^. The RNA/Zn^2+^ complex was fully able to catalyse the aldolase reaction and achieved a substantial rate 4300-fold above the background reactivity [22]. Hence, despite the fact that RNA molecules on their own might fall short of catalysing metabolic reactions, they are able to support such reactions by binding a metal catalyst. Interestingly, not only RNA, but also simple peptide motifs, are able to bind metals. The property of specific metal-binding peptide sequences has been extensively investigated, as peptide–metal complexes have a high potential as catalysts in synthetic organic, environmental and protein chemistry [38,39].

The hypothesis that RNA- and/or peptides bound to metal ions could have functioned as early enzymes is attractive in many ways. First, the origin of such a function does not require the establishment of a complex structure *ab initio*; only a moderate binding affinity of RNA or a short peptide would suffice to provide the starting point for evolutionary selection. Secondly, iron and other metals not only catalyse or support a single reaction within glycolysis and the PPP, but also catalyse multiple reactions and stabilize their sugar phosphate intermediates [27]. A simple metal-binding RNA or peptide structure could thus support multiple reactions in parallel, providing a starting point for positive selection of a functional entity.

Which advantages could the binding of a metal through an RNA or simple peptide provide? First, most of the metal ions only possess average water solubility, thus binding them would increase their accessibility. Secondly, despite iron being abundant in the Archaean sea, other metal ions such as molybdenum or zinc were not as frequent; also iron itself became limiting upon oxidation to the insoluble Fe(III) upon the great oxygenation event [28–35]. The affinity for RNA or peptides could thus increase the local concentration of a given metal ion, and prevent its loss through diffusion and/or precipitation.

**Figure 1:**
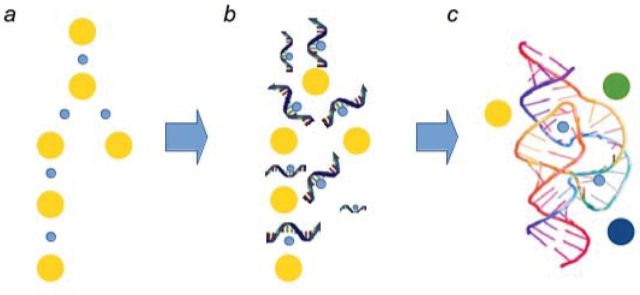
Did metal-based catalysis facilitate the formation of the first metabolic enzymes? (**a**) A precursor of glycolysis and the PPP was catalysed by ferrous iron and other metals found in the prebiotic oceans. (**b**) RNA molecules (and/or peptides?) bound the metal ions and in that way increased their accessibility and potentially their local concentration. (**c**) Positive selection of more sophisticated structures achieved specificity by preventing contact of the metal catalyst with some of the metabolites, and increasing the affinity for others.

Finally, RNA or peptide binding could increase the specificity of a reaction: metal ions such as Fe(II) act on a broad set of substrates. They are thus in many cases also catalysing unwanted reactions [40]. An example in modern cells is the Fenton reaction where ferrous iron produces superoxide from hydrogen peroxide, causing anything from oxidative stress up to fatal cellular toxicity [41]. To avoid Fenton chemistry, modern cells evolved a complex iron transport system and to circumvent the presence of ferrous iron in their cytoplasm [42]. Reflected in the primordial world, RNA structures could have prevented certain substrates from reaching the metal catalyst, and increased the affinity for others. A first enzyme would have been born (Figure 1).

Most modern enzymes of central carbon metabolism are not dependent on metallic co-factors. There are however examples which indicate that this could be the result of a later selection for modern metal-independent enzymes: ribulose-5-phosphate epimerase in *Escherichia coli* uses ferrous iron for its catalytic function [43], whereas the analogous ribulose-5-phosphate epimerase enzymes of higher organisms are not iron dependent [44]. These forms of the epimerase provide two advantages. First, they reduce the iron requirement of the species, and, secondly, they increase the oxidation-resistance of the PPP: the iron cofactor of the *E. coli* ribulose-5-phosphate epimerase renders this enzyme highly prone to peroxide oxidation, with the result that the bacterial species is sensitive to oxidants [43].

## Summary

The RNA world provides a sophisticated explanation for the origin of genetics, yet has problems in explaining the emergence of metabolism. The recent discovery of non-enzymatic, mainly metal-catalysed, glycolysis and PPP, however, supports a hypothesis that the first form of a metabolic network might have been shaped by environmental molecules that served as its first catalysts. A hybrid theory which considers the RNA world for the origin of genetics and prebiotic non-enzymatic (metal) catalysis for the origin of metabolism could help to solve problems associated with the origin of life. Importantly, this finding gives rise to a hypothesis that enzymes could have come into being as simple RNA and/or peptide molecules that possessed affinity for metal ions frequently found in the Archaean ocean.

## Abbreviations

PPP: pentose phosphate pathway
